# HIV-1 sequence evolution in vivo after superinfection with three viral strains

**DOI:** 10.1186/1742-4690-4-59

**Published:** 2007-08-23

**Authors:** Karolina Kozaczynska, Marion Cornelissen, Peter Reiss, Fokla Zorgdrager, Antoinette C van der Kuyl

**Affiliations:** 1 Laboratory of Experimental Virology, Department of Medical Microbiology, Centre for Infection and Immunity Amsterdam (CINIMA), Academic Medical Centre of the University of Amsterdam, Meibergdreef 15, 1105 AZ Amsterdam, The Netherlands; 2 Department of Internal Medicine, Division of Infectious Diseases, Tropical Medicine and AIDS, Academic Medical Centre of the University of Amsterdam, Meibergdreef 15, 1105 AZ Amsterdam, The Netherlands

## Abstract

With millions of people infected worldwide, the evolution of HIV-1 in vivo has been the subject of much research. Although recombinant viruses were detected early in the epidemic, evidence that HIV-1 dual infections really occurred came much later. Dual infected patients, consisting of coinfected (second infection before seroconversion) and superinfected (second infection after seroconversion) individuals, opened up a new area of HIV-1 evolution studies. Here, we describe the in-depth analysis of HIV-1 over time in a patient twice superinfected with HIV-1, first with a subtype B (B2) strain and then with CRF01_AE after initial infection with a subtype B (B1) strain.

The nucleotide evolution of *gag *and *env*-V3 of the three strains followed a similar pattern: a very low substitution rate in the first 2–3 years of infection, with an increase in synonymous substitutions thereafter. Convergent evolution at the protein level was rare: only a single amino acid in a *gag *p24 epitope showed convergence in the subtype B strains. Reversal of CTL-epitope mutations were also rare, and did not converge. Recombinant viruses were observed between the two subtype B strains. Luciferase-assays suggested that the CRF01_AE long terminal repeat (LTR) constituted the strongest promoter, but this was not reflected in the plasma viral load. Specific real-time PCR assays based upon the *env *gene showed that strain B2 and CRF01_AE RNA was present in equal amounts, while levels of strain B1 were 100-fold lower.

All three strains were detected in seminal plasma, suggesting that simultaneous transmission is possible.

## Background

The overall rate of evolution of human immunodeficiency virus type 1(HIV-1) is the highest documented for viruses to date. Several mechanisms contribute to this phenomenon, amongst them the high error rate of the viral reverse transcriptase (RT), which lacks an 3'→5'exonuclease proofreading capacity, the short generation time, and the high rate of recombination between viral genomes. Recombination is facilitated by the average presence of three to four proviral genomes in the infected cell [1], combined with the template-switching ability of the viral RT [2]. Recombinant genomes are most easily spotted when different subtypes of HIV-1 are involved, but as recombination is typical in HIV replication, recombinant viruses are present in any infected persons. The rate of evolution, e.g. the rate of nucleotide substitution and recombination, of HIV-1 as governed by the viral RT is supposed to be more or less constant. However, selection factors, such as host immune pressure and the use of antiviral drugs influence the viral quasi-species so that there can be rapid outgrowth of only a limited number of viral genomes. The outcome of these evolution and selection processes is such that viruses at the end of the infection (AIDS stage) are clearly related, but distinct from the quasi-species that was present during the acute infection and from the viruses seen during the chronic phase of the infection. HIV-1 variation over time has been studied extensively in patients infected with single strains (e.g. see [3,4]). It has been suggested that HIV-1 evolution follows a similar pattern in most patients, whereby a period of linear increase in divergence and diversity is replaced by a stabilization of diversity, and finally by an evolutionary slowdown late in infection, accompanied by the appearance of CXCR4 using viruses [3,4]. Due to the availability of effective anti-viral treatment, the later stages of viral evolution are nowadays more difficult to study in vivo. Studies on HIV-1 evolution, mainly focussing on recombination events, in dually infected patients [5-11] and in patients coinfected with three HIV-1 strains [12,13] have also been performed. However, most studies suffer from a lack of samples (insufficient follow-up), and/or of a precise timing of the infections. Therefore, a more detailed description of how different HIV-1 strains present in the same host influence each other, except for the occurrence of recombination, is not available yet. We described earlier a Dutch patient who was twice superinfected with HIV-1 at identified time points; once with a subtype B virus, and once with CRF01_AE after initial infection with a subtype B strain [14]. Here we present an extensive follow-up of the HIV-1 quasi-species in this patient after triple infection, both in blood and in seminal plasma. The influence of infection with a second or third strain upon the evolution of the other strains was investigated in the *gag *and *env *genes, as well as was the frequency of convergent evolution. Biological clones were generated to estimate the occurrence of recombination. Virus production of the distinct strains in blood and seminal plasma was measured to see if, and to what extent, replication of the three strains continues or whether there is outgrowth of a single virus species. Continuous expression of all three strains was observed. LTR-luciferase experiments suggested that the CRF01_AE LTR has substantially higher promoter activity than the LTR's of both subtype B strains from this patient. This increased promoter activity was not reflected in plasma viral load differences, where strain B2 and CRF01_AE had similar copy numbers, while the strain B1 viral load was substantially lower.

## Methods

### Patient samples and HLA-typing

Patient H01-10366 is infected with three HIV-1 strains (in, or shortly before 2001 with subtype B (strain B1), in autumn 2002 with subtype B (strain B2), and in summer 2003 with CRF01_AE [14]). The patient was first demonstrated to be HIV-1-seropositive in March 2001 at the Municipal Health Service anonymous testing facility in Amsterdam, and referred for follow up to the Academic Medical Centre of the University of Amsterdam. Blood plasma samples were thereafter obtained at regular hospital visits of the patient. At a few time-points, PBMC's were collected using the BD Vacutainer^®^CPT™ system (Becton Dickinson, Plymouth, UK). Semen samples were collected at the same visits, and centrifuged for 20 minutes at 600 g to collect the seminal plasma used in the experiments.

HLA-typing of patient H01-10366 was routinely performed at Sanquin Diagnostiek (Amsterdam, The Netherlands) and the following results were obtained: HLA class I: A3, A32(19), B8, B62(15), and Cw3 (Cw4–8 were not tested); HLA class II: DRB1*12, DRB1*13, DRB3* positive, DQB1*03 and DQB1*06.

### Plasma viral load

Blood plasma HIV-1 RNA was measured using the VERSANT HIV-1 RNA 3.0 assay (bDNA) (Bayer Diagnostics Division, Tarrytown, NY), which has a detection level of 50 copies/ml. The HIV-1 viral load of the seminal plasma was determined with an in-house real-time PCR assay, with primers located in the HIV-1 *pol *gene. Primer/probe sequences were: upstream primer 5'TGC ATT YAC CATACC TAG T 3', downstream primer 5'ATT GCT GGT GAT CCT TTC CA 3', and probe 5'AAA CAA TGA GAC ACC AGG GAT TAG ATA 3'. The detection limit of this assay was 5 HIV-1 RNA copies per reaction.

### Viral strain-specific PCR assays

Although the PCR primers used in this study are able to amplify both HIV-1 subtype B and CRF01_AE, the efficiency with which the strains are detected in a mixed sample differs. Therefore, three additional strain-specific nested primer sets located at approximately the same positions in the *env *gene were developed to detect the B1, B2 and AE strains more accurately (for primer sequences see Table 1). Reverse transcriptase (RT) reactions were performed with AMV RT (Roche Applied Science, Indianapolis, IN) and the 3'outer primer.

**Table 1:** Primer and probe sequences

Primers	Primer sequence 5'-3'	
V3 evolution B1 virus		
5'tripleB1_1	GAA AAT TTC ACA GAC AAT GCT	1^st ^PCR
3'tripleB1_rt	TTA ATT TTG TAA CTA TCA GTT C	1^st ^PCR
5'tripleB1_2	TAA TAG TAC AGC TGA ATG CAT	Nested PCR
3'tripleB1_3	AGT GTT ATT CCA TTT TGT TAA	Nested PCR
V3 evolution B2 virus		
5'tripleB2_1	GAC AAT TTC ACA GAC AAT AAG	1^st ^PCR
3'tripleB2_rt	TTA ATT TTT CAA CTG TCT GAT T	1^st ^PCR
5'tripleB2_2	TAA TAG TAC AGC TGA AGA CAG	Nested PCR
3'tripleB2_3	AGC ATT ACC CCA TTC TAC TCC	Nested PCR
V3 evolution AE virus		
5'tripleAE_1	GAA AAT CTC ACA GAT AAT ACC	1^st ^PCR
3'tripleAE_rt	AGT GCT CTT TTA ATT TTT CAG	1^st ^PCR
5'tripleAE_2	CAT AAT AGT GCA CCT TAA TAA	Nested PCR
3'tripleAE_3	CCA TTT TGT TCT ATT AAT CTC	Nested PCR
Taqman strain specific assay B1		
5'B1/B2triple-taqman	TTA ATT GTA CAA GAC CCA GCA ACA	
3'B1triple-taqman	AAG GTT ACA ATG TGC TTG CCT TA	
B1triple-probe2rev	TCT CCT ATT ATT TCT CCT GTT GCA T	5'label 6-FAM
Taqman strain specific assay B2		
5'B1/B2triple-taqman	TTA ATT GTA CAA GAC CCA GCA ACA	
3'B2triple-taqman	ACT AAT GTT ACA ATG TGC CTT T	
B2triple-probe	TAA AAA ATG CTT TCC CTG GTC CCA TA	5'label 6-FAM
Taqman strain specific assay AE		
5'AEtriple-taqman	TCA ATT GTA CCA GAC CCT CTA AC	
3'AEtriple-taqman	TTG TTC TAT TAA TCT CAC AAT A	
AEtriple-probe	TAT AGA ATA CTT GTC CTG GTC CCA TA	5'label 6-FAM

### Viral strain-specific real-time PCR assays

To measure the viral copy number of each of the three strains independently in a single sample, three additional real-time PCR assays were developed. Primers and probes for the three strains, B1, B2, and CRF01_AE, were located at approximately the same positions in the V3 region of the HIV-1 *env *gene (for primer and probe sequences see Table 1). No cross-reaction was found between each specific primer and probe set with the other strains of patient H01-10366. The detection limits of the assays were 10 HIV-1 RNA copies per reaction.

### Generation of biological clones

Freshly phytohemagglutinin (bioTRADING Benelux, Mijdrecht, The Netherlands), glutamax and interleukin-2 (Proleukine) stimulated peripheral blood mononuclear cells (PBMC's), obtained from four healthy (HIV-1 negative) human donors, were combined and cultured in RPMI 1640 medium (Invitrogen Corporation, Carlsbad, CA) supplemented with antibiotics, L-glutamine and 15% heat-inactivated foetal calf serum for 3 days. CD8+ T cells were depleted after 2 days using the Dynabeads M-450 CD8 kit (Invitrogen Corporation, Carlsbad, CA). Different concentrations (10^4^, 2.5 × 10^4^, 4 × 10^4 ^6 × 10^4 ^cells/well) of PBMC's from the HIV-1 infected patient were cocultivated with 1 × 10^6 ^CD4+ T cells in the same medium in 96-wells plates for 21 and 28 days, respectively. Each 7 days culture supernatants were tested for the presence of p24 with an in-house antigen capture enzyme-linked immunosorbent assay (ELISA). At the same time, to propagate the culture, one-third of the cell culture was transferred to new 96-wells plates and fresh PHA, Il-2 stimulated CD4+ cells were added. Viruses were considered to be clonal if less than one-third of the microcultures are positive at a given cell number (Poisson distribution). HIV-1 clones were expanded and cultured [15]. After 7 days the clones were harvested. PBMC's and supernatant were cryopreserved at -150°C [16].

### RT-PCR of *gag *and *env*

A 804 nucleotide HIV-1 *gag *gene fragment, encompassing the complete p17 gene and the first part of p24, and a 264 nucleotide V3 sequence of the HIV-1 *envelope *gene were amplified by RT-PCR as described [17,18]. To amplify the whole of *gag*-p17 the 5'primers described by Cornelissen et al [17] were replaced with outer primer 5'GAC GCA GGA CTC GGC TTG CTG A 3', and nested primer 5'TCC TTC TAG CCT CCG CTA GTC AA 3' (the original 5'outer primer). Primers used are able to amplify both subtype B and CRF01_AE.

### PCR amplification of *vpr *and *vpu*

The complete *vpr *and *vpu *genes of the biological clones were amplified and completely sequenced as described [19].

### Cloning and sequencing

HIV-1 *gag *and V3 fragments were cloned with the TOPO TA cloning kit (Invitrogen, Carlsbad, CA, USA), and sequenced with the BigDye Terminator cycle sequencing kit (Applied Biosystems, Foster City, CA, USA). Electrophoresis and data collection are performed on an ABI PRISM 3100 genetic analyser (also from Applied Biosystems). The number of clones (n) for each virus strain per time point varied from n = 4 till n = 54, with an average of 10 clones per virus strain per time point. For 2002 three consecutive time points were sequenced and pooled in the analysis.

For the biological clones multiple primer sets were used to generate overlapping fragments that were directly sequenced as described above.

### Nucleotide distance calculation and phylogenetic analysis

Sequences were aligned with and without reference HIV-1 *gag, vpr*,*vpu *and *env*-V3 sequences [20] using ClustalW available in BioEdit Sequence Alignment Editor version 7.0.1 [21]. Recombination events between the B1 and B2 strains in gene fragments from the biological clones were identified from the nucleotide alignments. Nucleotide distances were estimated with the Tamura-Nei [22] distance with the gamma model. This model corrects for multiple hits, and takes into account the different rates of substitution between nucleotides and the inequality of nucleotide frequencies. The nucleotide composition of the HIV genome is quite different from other species, being A-rich and C-poor. The gamma shape parameter α for HIV-1 *gag *(α = 0.25) and *env*-V3 (α = 0.38) was taken from Leitner et al. [23].

Neighbour-Joining (NJ) trees based upon Tamura-Nei distances were constructed with the MEGA 3.1 software package [24], and 1000 bootstrap replicates were analysed. Bootstrap values ≥ 80 were considered significant. Additional phylogenetic analyses were done with the parallel version of MrBayes 3.1 [25], modified so that the program now uses the sprng library [26] to generate independent streams of random numbers. MrBayes3.1 was run at the SARA High Performance Computing Facilities [27]. Here, posterior probability values ≥ 0.8 were considered significant.

### LTR-constructs and luciferase-assays

The LTR region of the viral genome (from the biological clones or from plasma for subtype AE) was amplified by nested reverse transcription (RT)-PCR, with primer sets described earlier [28]. PCR products were cloned into pCRII-TOPO (Invitrogen Corporation, Carlsbad, CA) using the *BfrI*-site. Four clones from each strain; B1, B2, AE, X and B (LAI), were sequenced as described above. Subtype X is a novel HIV-1 subtype distantly related to subtype K, discovered recently in a single patient [29]. For strain B2, two sizes of LTR fragments were discovered of which the longer one contained a duplication of 23 nucleotides and was named B2_L(ong), while the shorter LTR was designated B2_S(hort). Sequences were aligned and transcription factor binding sites were identified with TFSEARCH [30] and Alibaba 2.1 [28,31,32].

A representative clone for each subtype was selected for subcloning into pBlue3'LTR, which is a Bluescript KS(+) plasmid containing a *XhoI-BglI *LAI 3'LTR fragment. Then, constructs were digested with *BseAI *and *BfrI *and the fragment (position -147 to +63 of the viral genome) was cloned into pBlue3'LTR-luc as described previously [28].

The cervix carcinoma cell line C33A was used in all luciferase experiments. Cells were grown in 2-cm^2 ^wells to 60%–70% confluency as described earlier [28,33] and transfected by the calcium phosphate method [34]. Mixtures contained 100 ng of different LTR-luciferase constructs (B1, B2_S, B2_L, AE, X and B(LAI)), 0.5 ng of pRL-CMV plasmid (Promega, Madison, WI) expressing *Renilla *luciferase as an internal control for transfection efficiency [33], and pBluescript in such a concentration that the total amount of DNA would always be 1000 ng. To test the activation of the promoters by tat, constructs were titrated with different concentrations of a tat-expressing plasmid (pTAT). Cells were cultured for two days and lysed in Passive Lysis Buffer (Promega, Madison, WI). Firefly and *Renilla *luciferase activities were determined with the dual-luciferase reporter assay (Promega, Madison, WI) as described previously [33]. The activity of different constructs was calculated as the ratio of the firefly and *Renilla *luciferase activities, and corrected for between-session variation [35].

## Results

### Detection of the three viral strains in blood and seminal plasma

**Figure 1:**
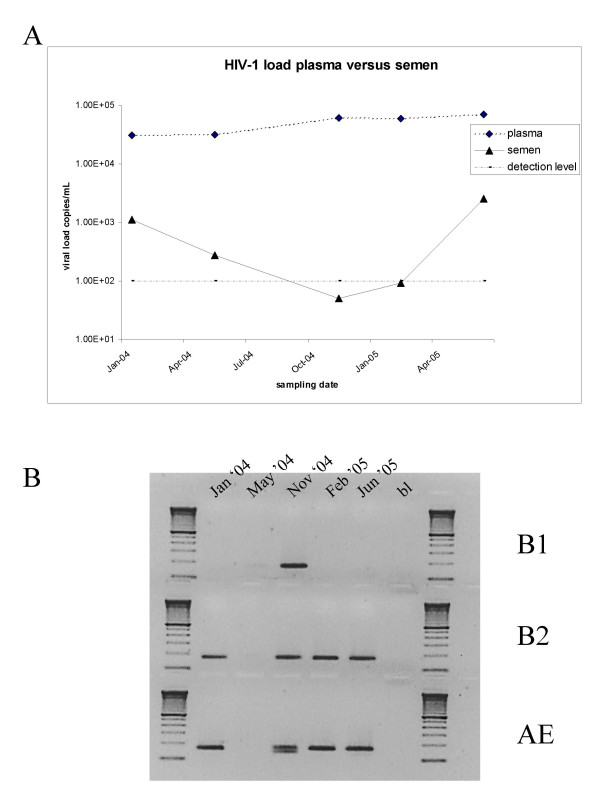
**Detection of the three viral strains over time**. (A) HIV-1 viral load in blood and seminal plasma. (B) Strain-specific RT-PCR detection of HIV-1 subtypes B (strains 1 and 2) and CRF01_AE in seminal plasma.

To verify the presence of the three viral strains over time in both blood and seminal plasma, three specific nested PCR primer sets were developed in the *env*-V3 region. Fig. 1 shows the overall viral load in blood and seminal plasma (panel A) and the detection of strains B1, B2 and CRF01_ AE in seminal plasma (panel B). In blood plasma, all three *env *fragments were detected by PCR amplification at all time-points, in line with the relatively high viral load (result not shown). In seminal plasma, however, the viral load was much lower and was sometimes even below the detection limit. In line with this, not all strains could be detected at every occasion (Fig. 1B), but over the course of the 1.5 years analysed here the patient was able to transmit any strain at some point. At all time points except one, at least two strains were simultaneously present. Interestingly, the *env *gene of the first infecting virus B1 was detected the least in seminal plasma.

**Figure 2:**
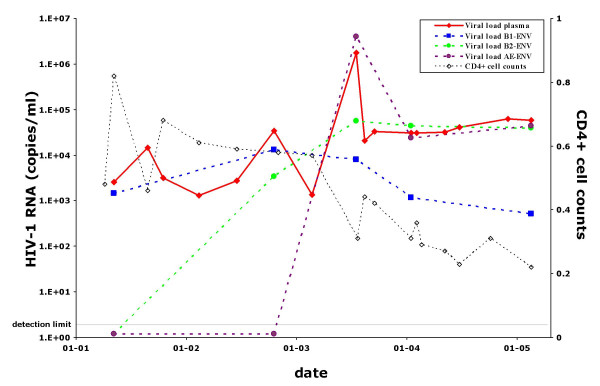
**Viral load of the three HIV-1 strains in blood plasma**. Real-time PCR analysis with specific primers and probes located in *env*-V3 was performed on sequential blood plasma samples of patient H01-10366. The plasma viral loads measured by real-time, strain specific PCR is shown, as well as the overall plasma viral load determined with the VERSANT HIV-1 RNA 3.0 assay, which is based upon the *pol *gene. CD4+ cell counts (× 10^9^) measured at the same time points are also shown.

To determine the contribution of each viral strain to the total blood plasma viral load, three strain-specific real-time PCR assays were used to amplify a fragment of *env-*V3 of strains B1, B2 and CRF01_AE in sequential plasma samples of patient H01-10366 (Fig. 2). As expected, sequences of strain B2 were not detected until the B2 superinfection moment, and the CRF01_AE sequences were similarly not detected until the CRF01_AE superinfection moment. At the latter time-point, the very high plasma viral load was mainly due to the newly infecting virus CRF01_AE. From the later time points, when three viruses are present in blood plasma, *env*-V3 sequences from strain B2 and CRF01_AE are present in more or less equal amounts (± 30.000 copies/ml), while strain B1 *env*-V3 sequences form a minority (less than 300 copies/ml). CD4+ cell counts are stable after superinfection with strain B2, but rapidly decrease after the second superinfection with CRF01_AE (Fig. 2).

### Analysis of biological clones

To assess the occurrence of recombination between the three strains, a total of 20 biological clones were generated corresponding to three time points (time point 1 = January 2004, time point 2 = February 2004, time point 3 = November 2004, approximately 6, 7, and 15 months after triple infection). Of these 20 clones, three were completely sequenced, while the structure of the other 17 was roughly analysed by amplifying and sequencing LTR, *gag*, *vpr*, *vpu*, and *env*-V3 fragments (Table 2). Two clones, 2301#12 and 2602#1, of which the former was completely sequenced, appeared to contain a complete strain B2 virus, while the other 18 clones were all recombinants between B1 and B2 virus sequences. No full-length B1 or CRF01_AE viruses found, nor were any CRF01_AE sequence fragments detected in the clones. Recombination between the strain B1/B2 viruses was found at different sites; the analysis of 10 clones suggested that recombination occurred between the *gag *and the *vpr *genes, in one virus recombination occurred in the *gag *gene (between p17/p24), and in another virus recombination was found in the *vpr *gene. Six clones showed a more complex pattern of recombination, with multiple crossover sites being present (Table 2). In general in the recombinants, the genome composition was such that the 5' end of the virus originated from strain B1, while the 3'half of the viruses corresponded to strain B2, except of course for the 3'LTR, which belonged to B1 again.

**Table 2:** Genomic organization of 20 biological clones

**Date**	**Clone**	**LTR**	**Gag**	**Vpr**	**Vpu**	**V3 env**
23.01.04	2301#1	**B1**	**B1**	B2	B2	B2
	2301#2	**B1**	**B1**	B2	B2	B2
	2301#3	**B1**	**B1**	B2	B2	B2
	2301#4	B2	**B1**	B2	B2	B2
	2301#5	**B1**	**B1**	B2	**B1**	**B1**
	2301#6	**B1**	**B1**	B2	B2	B2
	2301#7	**B1**	**B1**	B2	**B1**	B2
	2301#8	**B1**	**B1**	B2	B2	B2
	2301#9	**B1**	**B1**	B2	B2	B2
	2301#10	**B1**	**B1**	B2	B2	B2
	2301#11	**B1**	**B1**/B2	B2	B2	B2
	2301#12	B2	B2	B2	B2	B2
	2301#13	**B1**	**B1**	**B1**/B2	B2	B2
	2301#14	**B1**	**B1**	B2	B2	B2
	2301#15	**B1**	**B1**	B2	B2	B2
26.02.04	2602#1	B2	B2	B2	B2	B2
	2602#2	**B1**	**B1**	B2	**B1**/B2	**B1**
03.11.04	0311#1	**B1**	**B1**	B2	B2	B2
	0311#2	**B1**	**B1**	B2	**B1**/B2	**B1**
	0311#3	**B1**	**B1**	B2	B2	**B1**

**Figure 3:**
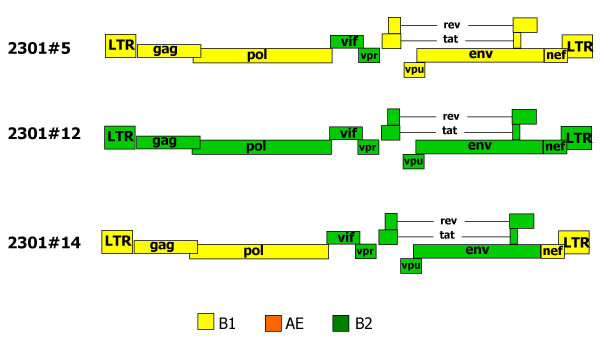
**Genomic organization of three completely sequenced biological clones from the January 2004 time point**. Clone 2301#12 contained a complete strain B2 virus, while clones 2301#5 and 2301#14 were strain B1/B2 mosaics. CRF01_AE sequences were not found.

Of the completely sequenced virus clones, the genomic structure is shown in Fig. 3. Clone 2301#5 was found to be almost completely composed of strain B1 sequences, except for a small part in the middle of the genome encompassing the *vif *and *vpr *genes, which originated from strain B2. Clone 2301#12 contained a complete strain B2 virus. Clone 2301#14 was a more complex recombinant virus where recombination did occur once between the *pol *and *vif *genes, and again between the *env *and *nef *genes.

Because no CRF01_AE sequences were found amongst the biological clones, the presence of CRF01_AE DNA in the PBMC samples used in the biological cloning procedure was analysed with PCR primers specific for CRF01_AE. Indeed, CRF01_AE *env*-V3 sequences were present in the preparations (not shown); suggesting the deficiency of the clones is not explained by the absence of viral DNA.

### Evolution of *gag*: nucleotide distances

**Figure 4:**
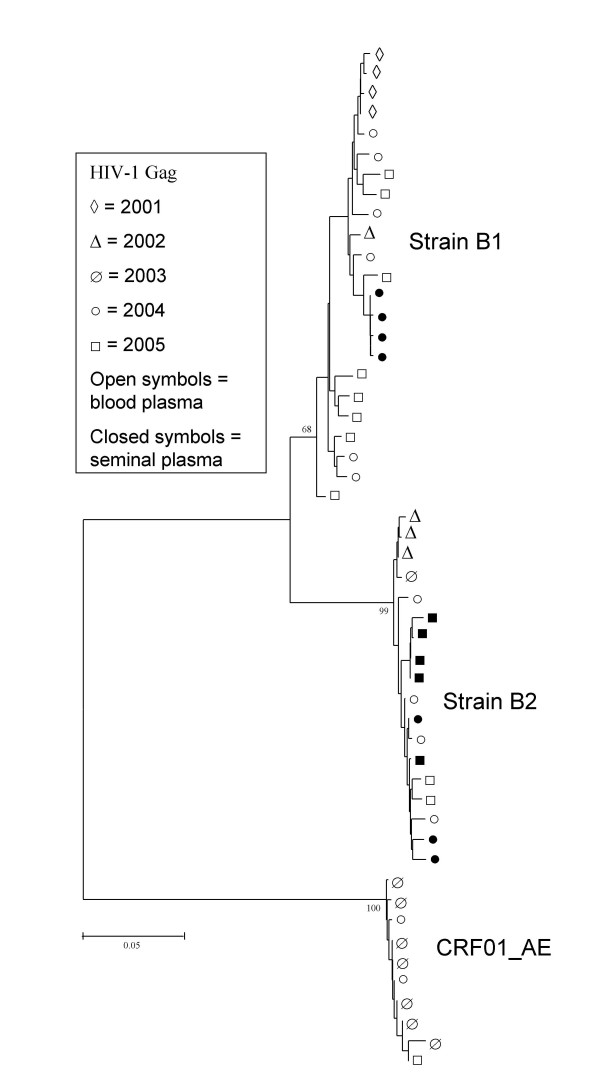
**Phylogenetic analysis of HIV-1 *gag *sequences**. NJ tree of HIV-1 *gag *nucleotide fragments obtained from blood and seminal plasma collected in 2001–2005. Distances were calculated with the Tamura-Nei method using the gamma model with α = 0.25, and 1000 bootstrap replicated were analysed. The three separate clusters comprised of strains B1, B2, and CRF01_AE are indicated. A representative sequence set was used to draw the phylogenetic tree.

Having three distinguishable virus strains in one patient is a great opportunity to learn whether or not the nucleotide evolution of a single virus is influenced by the presence of other virus strains. Therefore we amplified, cloned and sequenced *gag *gene fragments from consecutive time points for the B1, B2 and AE viral strains using generic PCR-primers, and calculated their overall diversity (= nucleotide distance) per year in both blood and seminal plasma. Mean nucleotide distances for the *gag *gene fragments are summarized in Table 3. From this table it is clear that nucleotide variation in blood plasma follows a similar pattern for viral strains B1 and B2, although strain B2 has a relatively high amount of synonymous variation in the year of initial infection (2002). In approximately the first 2–3 years of infection, overall nucleotide variation is low for both strains. After this period (in 2004 for B1, and in 2005 for B2), mean nucleotide differences start to rise. This rise is almost completely accounted for by an increase in synonymous substitutions. The amount of non-synonymous substitutions does not differ significantly over the years in both strains. A phylogenetic NJ tree based upon *gag *sequences is shown in Fig. 4. From this tree, the low level of evolution of HIV-1 *gag *in this patient is also obvious from the short branch lengths. A similar phylogenetic tree was obtained with a Bayesian approach.

**Table 3:** Mean nucleotide distances within the gag gene over time in blood and seminal plasma

	B1 strain			B2 strain			Subtype AE		
Year	Tamura-Nei^a^	Syn^b^	Nonsyn^c^	Tamura-Nei^a^	Syn^b^	Nonsyn^c^	Tamura-Nei^a^	Syn^b^	Nonsyn^c^

2001 blood	0.008 ± 0.001	0.011 ± 0.004	0.006 ± 0.001	-	-	-	-	-	-
2002 blood	0.007 ± 0.001	0.012 ± 0.003	0.005 ± 0.001	0.009 ± 0.002	0.022 ± 0.006	0.005 ± 0.001	-	-	-
2003^d ^blood	-	-	-	-	-	-	0.003 ± 0.001	0.002 ± 0.002	0.003 ± 0.001
2004 blood	0.022 ± 0.004	0.071 ± 0.012	0.006 ± 0.002	0.008 ± 0.002	0.008 ± 0.004	0.008 ± 0.002	0.004 ± 0.002	0.006 ± 0.006	0.003 ± 0.002
2005 blood	0.021 ± 0.003	0.051 ± 0.009	0.010 ± 0.002	0.025 ± 0.005	0.060 ± 0.014	0.011 ± 0.003	-^e^	-	-
2004 semen	0.008 ± 0.002	0.011 ± 0.003	0.006 ± 0.002	0.002 ± 0.001	0.006 ± 0.004	0.001 ± 0.001	-^e^	-	-
2005 semen	0.007 ± 0.002	0.017 ± 0.005	0.003 ± 0.001	0.008 ± 0.002	0.007 ± 0.004	0.008 ± 0.002	-^e^	-	-

^a^: Tamura-Nei distance with gamma-parameter α = 0.25. Standard errors were estimated by the bootstrap method with 500 bootstrap replicates each.

^b ^and ^c^: Nei-Gojobori method, p-distance, syn= synonymous, nonsyn= nonsynonymous

^d ^Nucleotide differences of strains B1 and B2 were not calculated in 2003, because only B1/B2 or B/AE recombinant sequences were retrieved.

^e ^Nucleotide distances were not calculated for subtype AE in 2005 (blood plasma), 2004 and 2005 (seminal plasma) as no AE fragments were amplified by the generic primers from these samples.

Mean nucleotide distances in seminal plasma are lower than in blood plasma for virus strains B1 and B2. This is probably correlated with the low HIV-1 copy number in seminal plasma compared with the blood compartment (Fig. 1). Phylogenetic analysis of blood plasma and seminal plasma derived HIV-1 *gag *sequences suggest that there are no semen specific sequences and that compartmentalization does not occur in the seminal compartment (Fig. 4). Seminal plasma *gag *sequences cluster together with blood plasma sequences from the corresponding time points for both strains. This sampling time-related clustering was also seen for the *gag*-sequences obtained from the biological clones (not shown).

### Evolution of *env*-V3: nucleotide distances

**Figure 5:**
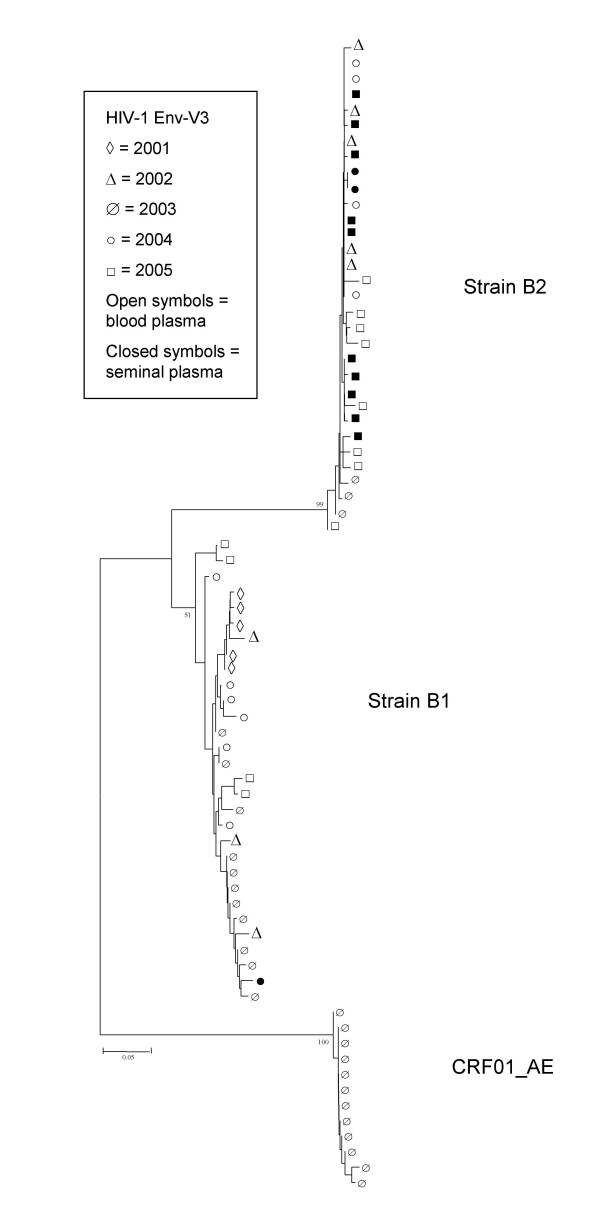
**Phylogenetic analysis of HIV-1 *env*-V3 sequences**. NJ tree of HIV-1 *env*-V3 nucleotide fragments obtained from blood and seminal plasma collected in 2001–2005. Distances were calculated with the Tamura-Nei method using the gamma model with α = 0.38, and 1000 bootstrap replicated were analysed. The three separate clusters comprised of strains B1, B2, and CRF01_AE are indicated. A representative sequence set was used to generate the phylogenetic tree.

Mean nucleotide distances per year of the *env*-V3 region of the viral genome of strains B1, B2, and CRF01_AE are shown in Table 4. Overall nucleotide distances in blood plasma slowly rise over the years for all three strains. This rise is mostly accounted for by an increase in synonymous substitutions, while non-synonymous nucleotide distances are more or less constant throughout the period investigated. Mean nucleotide distances were also calculated for the viral population of strains B1 and B2 amplified from seminal plasma (Table 4), and were found to be similar to the blood plasma values, despite the much lower viral load in seminal plasma. For strain B1, no *env*-V3 fragments could be amplified from the 2005 seminal samples (Fig. 1). Figure 5 shows an NJ tree based upon V3 nucleotide fragments from 2001–2005 from both blood and seminal plasma. It is obvious from this tree that there is very little sequence evolution in V3 in this patient, as indicated by the short branch lengths. Sequences did not cluster according to year or compartment. Both phylogenetic methods (NJ and Bayesian analysis) yielded similar trees.

**Table 4:** Mean nucleotide distances within env-V3 over time in blood and seminal plasma

	B1 strain			B2 strain			Subtype AE		
Year	Tamura-Nei^a^	Syn^b^	Nonsyn^c^	Tamura-Nei^a^	Syn^b^	Nonsyn^c^	Tamura-Nei^a^	Syn^b^	Nonsyn^c^

2001 blood	0.010 ± 0.002	0.011 ± 0.003	0.008 ± 0.002	-	-	-	-	-	-
2002 blood	0.017 ± 0.004	0.014 ± 0.005	0.012 ± 0.003	0.006 ± 0.002	0.010 ± 0.004	0.004 ± 0.002	-	-	-
2004 blood	0.012 ± 0.004	0.012 ± 0.007	0.010 ± 0.004	0.005 ± 0.003	0.000 ± 0.000	0.006 ± 0.003	0.009 ± 0.002	0.010 ± 0.002	0.008 ± 0.002
2005 blood	0.028 ± 0.009	0.050 ± 0.021	0.015 ± 0.006	0.022 ± 0.006	0.040 ± 0.014	0.014 ± 0.004	0.010 ± 0.003	0.021 ± 0.003	0.005 ± 0.002
2004 semen	0.009 ± 0.004	0.017 ± 0.012	0.006 ± 0.003	0.008 ± 0.003	0.013 ± 0.009	0.005 ± 0.002	-^d^	-	-
2005 semen	-^d^	-	-	0.010 ± 0.003	0.017 ± 0.010	0.006 ± 0.002	-^d^	-	-

^a^: Tamura-Nei distance with gamma-parameter α = 0.38. Standard errors were estimated by the bootstrap method with 500 bootstrap replicates each.

^b ^and ^c ^Nei-Gojobori distance method, p-distance, syn = synonymous, nonsyn= nonsynonymous.

^d ^Nucleotide distances were not calculated for strain B1 in 2005 (seminal plasma) and subtype AE in 2004 (seminal plasma) and 2005 (seminal plasma) as the generic primers did not amplify B1 or AE fragments from these samples, and the products generated with specific primers were too short to conduct evolution studies.

### Evolution of *gag *and *env*: CTL-epitopes

Escape from CTL pressure, or reversion of escape mutations, is one of the main driving forces in HIV evolution [36-38]. We therefore set out to examine mutations in CTL epitopes of this triple infected patient, and to investigate whether or not escape (or reversal) occurs in more than one virus strain. Visual inspection of the translated gag amino acid alignment suggested only a single site displaying convergent evolution in both subtype B viruses: amino acid 41 of gag p24 showed a S→T substitution which was found in none of the early viruses, but in over 90% of the 2005 viruses of both the B1 and B2 strains. The S→T substitution was not seen in the subtype AE sequences. Serine-41 belongs to a CTL epitope that is strongly reactive in ethnic Africans, but has not been associated with a specific HLA type [39]. Ser-41 is not one of the major phosphorylation sites of the HIV CAp24, which are Ser-109, Ser-149, and Ser-178, thus probably allowing the substitution observed [40]. However, replacing Ser-41 with Ala-41 delayed replication of the mutated virus in vitro [40], suggesting that it affects viral fitness. As the S→T substitution is observed in both strains B1 and B2, pressure from CTL's directed at this epitope is likely to be high in this patient. In contrast, the CRF01_AE virus did not react to this hypothetical immune pressure, and did not replace Ser-41 over two years of infection. Ser-41 is also part of a HIV-1 CD4+ T-cell epitope [41,42]. The peptide SPEVIPMFSALSE (p24_33–45_, Ser-41 is underlined) was found to bind to several HLA-DR molecules [42]. This suggests that CD4+ T cell responses could also be responsible for shaping viral evolution in this patient.

According to the HLA type of our patient, 6 epitopes could be recognized in the gag and the env fragments obtained. These epitopes together with the deduced amino acid sequence of the viral strains are listed in Table 5. The p24 epitope mentioned above for which no associated HLA type is known, but for which a viral reaction is seen in this patient, is also included in Table 5. For the other epitope in p24, all three viruses have a possible escape mutation already at the earliest time point, and no changes are seen over time. Two B8 restricted epitopes are apparent in gag p17. All three viruses have at least one mutation from the consensus sequence of the epitope, but in two instances a reversal to a more ancestral state is seen (in B1 by substituting V→I in EVKDTKEAL, and in B2 by substituting F→Y in ELKSLFNTV), suggesting that no CTL pressure is exerted upon these sequences. None of these substitutions occurs in any other strain. At the first gag p17 ^aa(18–28) ^epitope, restricted by the HLA-A3 allele, mutations are seen in both the B1 and the B2 strains. This epitope has been determined to be the most dominant gag CTL-epitope in Caucasians in vivo [39], also because HLA-A3 has a high phenotypic frequency in Caucasians. However, mutations in strains B1 and B2 are different both at the start of the infection, although they involve the same amino acid residue, and after a number of years. CRF01_AE did not show any changes in this epitope, but had a different sequence from B1 and B2 at the time of infection (with the derived C-terminal amino acid being a Q instead of an R (B1), or an S (B2)).

**Table 5:** Predicted CTL epitopes in HIV-1 *gag *and *env*-V3 (according to the patients HLA type) and their evolution

**Protein, position**	**CTL epitope**	**HLA-I type**	**Subtype B1 2001**	**Subtype B2 2002**	**Subtype AE 2003**
**Gag p17, 18–28**	KIRLRPGGK or RLRPGGKKK	A3	KIRLRPGG**K**KR* K→R (42%) in 2005	KIRLRPGGKK**S** S→R (100%) in 2005	KIRLRPGGKKQ, no changes over time
**Gag p17, 74–82**	ELRSLYNTV	B8	ELKSLYNT**V**, 50% **V**→I in 2005	ELKSL**F**NTV, 67% **F**→Y in 2005	ELKSLYNTV, no changes over time
**Gag p17, 93–101**	EIKDTKEAL	B8	E**V**KDTKEAL, 83% **V**→I in 2005	DVKDTKEAL, no changes over time	EILDTKEAL, no changes over time
**Gag p24, 8–21**	GQMVHQAISPRTLN	A3- supertype Cw3	GQMVHQPISPRTLN, no changes over time	GQMVHQPISPRTLN, no changes over time	GQMVHQPVSPRTLN, no changes over time
**Gag p24, 41–60**	SALSEGATPQDLNTMLNTVG	unknown	**S**ALSEGATPQDLNTMLNTVG 92% **S**→T in 2005	**S**ALSEGATPQDLNTMLNTVG 96% **S**→T in 2005	SALSEGATPQDLNMMLNIVG, no changes over time
**Env-V3, 296–305**	CTRPNNNTRK	A3	CTRPSNNTRK, no changes over time	CTRPSNNTRK, no changes over time	CTRPSNNTRT, no changes over time
**Env-V3, 308–322**	RIQRGPGRAFVTIGK	A3	SIHIAPGRAFYATGE, no changes over time	SIHMGPGKAFFTTGE, no changes over time	SIHMGPGQVFYRTGD, no changes over time

* Underlined amino acids are deviations from the consensus epitope sequence. Changes over time are marked in bold.

Two HLA-A3 epitopes are predicted in env-V3. All three HIV-1 strains have mutations in these motifs at the start of the infection, and no changes over time (from the years 2001 to 2005) are seen in any strain (Table 5).

### LTR promoter activity

**Figure 6:**
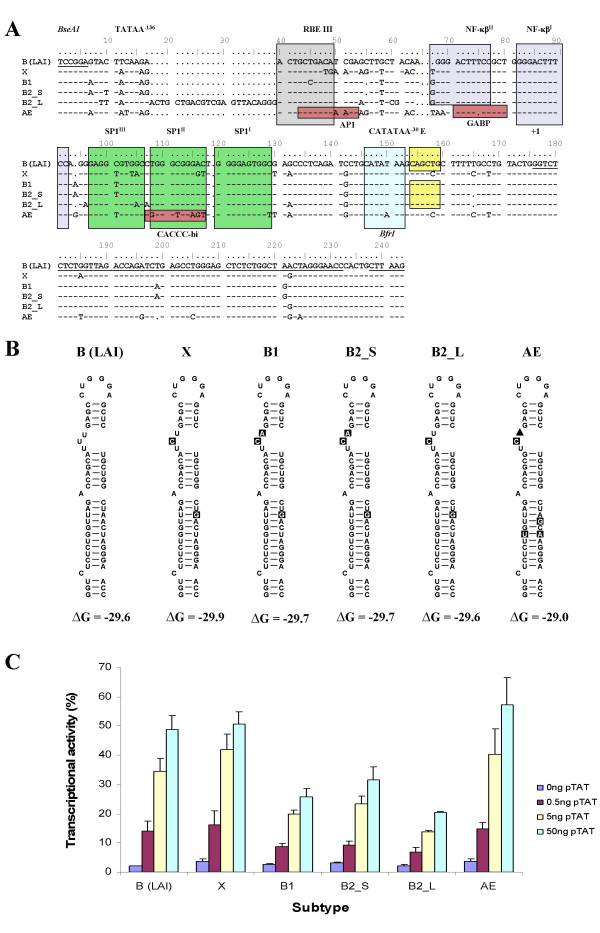
**Structure and activity of LTR sequences from strains B1, B2, and subtype AE**. **A**. Partial LTR sequence of subtypes B1, B2_S, B2_L, AE and X. The LTR region, spanning position -147 to +67 of reference strain B (LAI) is shown at the top. Dashes indicate nucleotides that are identical in subtype B(LAI), gaps are represented by dots. Restriction sites used in cloning are italicised and underlined. Boxes indicate motifs possibly involved in promoter function [28]. Subtype AE has three unique transcription factor binding sites: an AP1 motif, a GABP motif and a CACCC box-binding factor motif [46,47]. The TAR hairpin sequence (position 176–232) is underlined. **B**. Structure of the TAR RNA secondary structure in different HIV-1 subtypes, using the structure of subtypes B(LAI) and X [29] as references. Nucleotide differences between the strains are boxed and nucleotide deletions are indicated by (black triangle). A detailed phylogenetic analysis of HIV-1 subtype B TAR sequences has been described previously [48,49]. **C**. Transcriptional activity of the HIV-1 LTR promoter sequences from strains B(LAI), X, B1, B2_S, B2_L, and AE. Transcriptional activity was tested in the presence of increasing concentrations of Tat. The value is the average of four independent measurements; the standard deviation is indicated.

Promoter activity of the LTR sequence of strains B1, B2 and CRF01_AE from patient H01-10366 was analysed with a luciferase-assay. Aligned LTR sequences are shown in Fig. 6A, together with those from controls B(LAI) and subtype X (chosen because its TAR hairpin is identical to that of B2_L, Fig. 6B). Fig. 6C shows the transcriptional activity of the 6 LTR constructs, in the presence of different concentrations of tat. It is clear that the LTR of subtype AE has a comparable activity to that of the controls B(LAI) and subtype X, but that the activity of the three subtype B constructs of patient H01-10366 is much lower. The B2_L construct has the lowest activity of all, suggesting that the 23 nt duplication is decreasing promoter activity. This longer LTR was found in two of the three biological clones that contained a B2 LTR (2301#12 and 2602#1); the shorter LTR was only seen once (in clone 2301#4).

## Discussion

Having a patient twice superinfected with HIV-1 provides a unique opportunity to study the evolution of three distinct HIV strains in a shared in vivo environment. We have analysed different aspects of the viruses of patient H01-10366, including the plasma viral load of each strain over time, the presence of each strain in seminal plasma, the rate of nucleotide evolution, the occurrence of recombination, and of possible convergent CTL escape mutations. Finally, we have analysed the strength of the viral LTR's as promoter sequences in luciferase-assays.

Interestingly, all three virus stains, two subtype B strains named B1 and B2 and CRF01_AE, remain detectable in the plasma until at least two years after the second superinfection with CRF01_AE in 2003. In blood plasma, the viral loads of strain B2 and CRF01_AE are comparable, and approximately 100× higher than that of strain B1, the first infecting virus. In seminal plasma, the average total viral load is 100× lower than in blood plasma; at a single time point HIV-1 is undetectable by PCR. Here, the virus strains have only been detected qualitatively, but the overall picture is similar: the B1 strain is sometimes undetectable, suggesting it has a low copy number, while the B2 and AE strains are always detectable (except for the single negative sample), implying a much higher copy number. The almost continuous presence of all three viral strains in seminal plasma implies that this triply infected patient is able to transmit multiple strains at most time points.

The LTR-luciferase assays suggested that the LTR from CRF01_AE has a much higher activity in vitro than either subtype B LTR, but this difference is not reflected in the in vivo viral load in blood plasma. It is possible that the cervix carcinoma cell line used in the in vitro assays does not reflect the in vivo situation due to differences in the availability or concentration of transcription factors. Previous work also showed that the CRF01_AE LTR is much more potent in vitro than LTR's from subtype B [28]. Early after seroconversion, patients infected with CRF01_AE also show a three times higher viral load than those infected with subtype B, although viral load differences decrease later on [43]. Possibly, CRF01_AE cannot replicate to its full extent after early infection due to decreasing levels of available CD4+ T cells. Interestingly, some strain B2 viruses contained a repeat-like insertion of 23 bp in the LTR that decreased the in vitro promoter activity, but did result in viable viruses as it was found amongst the biological clones. In the LTR sequence of CRF01_AE, the most active promoter of the three viruses in the in vitro assays, three transcription factor binding motifs were different from the subtype B LTR's. One of the NF-κB sites is mutated to a GABP site, an SP1 site is mutated into a CACCC binding motif, and a novel AP1 site overlaps the RBE III site. However, none of these changes were present in the subtype B (LAI) and X LTR's, which were similarly active in vitro.

In this triple HIV-1 infected patient, copy numbers of the first virus, strain B1, decrease sharply after the second superinfection, suggesting that the superinfections could have been facilitated by an initial infection with a less fit virus. Another explanation for the apparent disappearance of strain B1 can be found in the analysis of the biological clones generated from samples postdating the second superinfection. Of the 20 clones examined, 18 were found to be recombinants between the B1 and B2 strains, with 14 clones having a B2 *env *gene sequence and only four clones having a B1 *env *gene sequence. If indeed B1/B2 recombinant viruses with mainly strain B2 *envelope *sequences have by then become the major virus population in blood, assays targeting the *env *gene will underestimate the level of B1 sequences. An assay targeting e.g. the *pol *gene might well overestimate strain B1, and give lower values for strain B2 copy numbers.

No CRF01_AE sequences were detected amongst the biological clones, neither as full-length viruses nor as recombinant viruses. Other experiments showed that CRF01_AE DNA was present in the patients PBMC's and that CRF01_AE RNA could be detected at high levels in blood plasma. If CRF01_AE does not grow in our donor PBMC's as well as the subtype B strains, more biological clones should be analysed to optimize the detection of this virus. On the other hand, biological clones were generated using techniques that are probably optimized for HIV-1 subtype B, suggesting that modifications to the protocol are needed to increase the likelihood of obtaining CRF01_AE clones. The absence of AE/B recombinant viruses could be due to the low frequency of recombination between subtype B and CRF01_AE. Although multiple subtype B/CRF01_AE recombinant viruses are circulating in Asia (see e.g. [44]), the in vitro recombination rate between subtype B and CRF01_AE is 9-fold lower than the intrasubtype recombination rate, mainly due to mismatches in the dimerization initiation signal (DIS) [45]. For subtype C and CRF01_AE, which have an identical DIS, the intersubtype recombination rate was only two-fold lower than the intrasubtype rate [45]. So, to detect any recombination between subtype B and CRF01_AE in this patient, many more clones need to be analysed due to its estimated minor frequency. Unfortunately, biological cloning using patient H01-10366 PBMC's was very inefficient in our hands, and sample limitations disabled further efforts.

Nucleotide and deduced amino acid sequences of the *gag *and the *env *gene were also analysed over time in this patient. Viral strains B1 and B2 followed a more or less similar trajectory, whereby nucleotide substitutions were low in the first 2–3 years, after which the synonymous substitution rate increased. Follow-up for CRF01_AE was much shorter, but no deviation from the subtype B pattern was evident. The nonsynonymous substitution rate remained rather constant over the years. This low nonsynonymous evolution rate was connected to another remarkable aspect of HIV-1 in this patient: the virtual lack of CTL-epitope evolution. Gag and env epitopes, as taken from the Los Alamos Database according to the patients HLA type, were studied longitudinally. No changes were seen in any env-V3 epitope. A convergent change in the gag p24 epitope SALSEGATPQDLNTMLNTVG was seen in strain B1 and strain B2. Here, the N-terminal S was changed into a T after 3–4 years of evolution, suggesting intensive CTL pressure. However, as we did not measure actual CTL responses in this patient, and this serine is also part of a CD4 epitope, it is unclear if the escape is really due to CTL effects. Reversal of CTL escape mutations in part of the viral population was seen in two *gag *epitopes in strain B1 and B2. In the *gag *p17 epitope ELRSLYNTV, 67% of the B2 strain reversed its escape mutation F to wild type Y after three years of infection. However, in strain B1, 50% of the viral population contained after four years of evolution a C-terminal I instead of the V present in both the consensus epitope and in strain B2. Also, the second epitope in *gag *p17 a change was seen in strain B1 (83% V→I), but this amino acid remained a V in strain B2. So, for the changes in *gag *p17, it is unclear whether the (absence of) CTL pressure has introduced them, especially as we did not examine the CTL response of patient H01-10366.

Overall, the different HIV-1 strains found in patient H01-10366 seem to influence each others evolution only minimally, except for excessive recombination between the subtype B strains. It is striking that the later arriving viruses (strain B2 and CRF01_AE) replicate at much higher levels in blood compared with the first infecting virus B1. Because the assays are targeted at *env*-V3, and many recombinant viruses were found to contain a 5'end of one strain and a 3'genomic part of the other subtype B strain, it is possible that B2 copy numbers are apparently increased because of the replication of a B1/B2 recombinant virus with a B2 *env *gene. In both subtype B strains mutations (either forward or reverse) are observed, but no changes are seen in CRF01_AE. This suggests that immune pressure is waning later in infection, and coincides with clinical progression in the patient after the second superinfection. Decreasing CD4+ cell counts at that time are soon followed by the initiation of antiretroviral therapy. Surprisingly, the first superinfection did not result in lowering of the CD4+ cell numbers. This suggests that the immune system of the patient was able to cope with a second HIV-1 subtype B virus, but not with the more distantly related CRF01_AE variant. At present, antiretroviral therapy is successful in this patient, and the plasma viral load has become undetectable.
